# Foraging behaviour and habitat use during chick-rearing in the Australian endemic black-faced cormorant (*Phalacrocorax fuscescens*)

**DOI:** 10.1242/bio.060336

**Published:** 2024-05-16

**Authors:** Thomas Cansse, Luc Lens, Grace J. Sutton, Jonathan A. Botha, John P. Y. Arnould

**Affiliations:** ^1^ School of Life and Environmental Sciences, Deakin University, Burwood 3125, Australia; ^2^ Terrestrial Ecology Unit, Ghent University, Ghent 9000, Belgium

**Keywords:** Seabird, Cormorant, Foraging behaviour, Habitat use, Diet, Bass Strait

## Abstract

Despite its wide distribution, relatively little is known of the foraging ecology and habitat use of the black-faced cormorant (*Phalacrocorax fuscescens*), an Australian endemic seabird. Such information is urgently required in view of the rapid oceanic warming of south-eastern Australia, the stronghold of the species. The present study used a combination of opportunistically collected regurgitates and GPS/dive behaviour data loggers to investigate diet, foraging behaviour and habitat-use of black-faced cormorants during four chick-rearing periods (2020-2023) on Notch Island, northern Bass Strait. Observed prey species were almost exclusively benthic (95%), which is consistent with the predominantly benthic diving behaviour recorded. Males foraged at deeper depths than females (median depth males: 18 m; median depth females: 8 m), presumably due to a greater physiological diving capacity derived from their larger body size. This difference in dive depths was associated with sexual segregation of foraging locations, with females predominantly frequenting shallower areas closer to the coastline. These findings have strong implications for the management of the species, as impacts of environmental change may disproportionally affect the foraging range of one sex and, thereby, reproductive success.

## Teaser

**Summary:** In black-faced cormorants, a little studied endemic Australian seabird, males and females exhibited different diving behaviour and exploited different areas. Thereby, environmental changes could disproportionally affect a single sex.

## INTRODUCTION

Resources, such as food, are often patchily distributed and can vary substantially in availability and abundance over time (MacArthur and Pianka, 1966; Mortelliti and Boitani, 2008). Consequently, searching for and obtaining food is a main factor driving the movements of animals through their habitat (McIntyre and Wiens, 1999; Pyke, 1984; Wilson et al., 2012). Top predators often target highly mobile prey species, and their spatial and temporal distribution, as well as their foraging behaviour, reflect this (Courbin et al., 2013; Fortin et al., 2015). Therefore, information on the movements and habitat-use of top predators is vital for determining the key habitats for their survival and reproductive success, as well as the factors driving their distribution (Jeltsch et al., 2013).

Due to its highly dynamic nature, the distribution and abundance of prey species in the marine environment are highly temporally and spatially variable (Carpenter-Kling et al., 2020; Choy et al., 2020; Croxall et al., 1985; Dänhardt and Becker, 2011; Weimerskirch, 2007). This is especially the case for pelagic prey species, which can have high abundances, but low predictability (Dänhardt and Becker, 2011; Gende and Sigler, 2006; Riverón et al., 2021). In contrast, benthic prey species are more predictable but, in general, less abundant (Elliott et al., 2009; Filatova et al., 2022; Litzow et al., 2004). Therefore, understanding how marine predators target different prey types, and which prey types they target, is central to a greater understanding of their foraging behaviour (Young et al., 2015).

Seabirds are a globally occurring, polyphyletic avian group that exploits marine habitats and are often positioned at the top of the food chain (Croxall, 1987; Schreiber and Burger, 2001). Due to their high trophic position, they are vulnerable to a myriad of factors and, as a group, are globally endangered (Croxall et al., 2012). During the breeding season, seabirds are central place foragers and the time they can spend, and distance they can range, on a foraging trip is limited by the fasting ability of their chicks and/or the amount of food the parents can carry (Chaurand and Weimerskirch, 1994; González-Solís et al., 2000). As foraging behaviour during the breeding season is strongly linked to reproductive success, data collected on foraging behaviour and habitat use at this time of the year is important for predicting possible effects of environmental changes on reproductive success and population trajectories (Grémillet and Boulinier, 2009; Lorentsen et al., 2019). However, while some species have been well studied, large knowledge gaps still exist for others, limiting our ability to predict their responses to environmental change (Bernard et al., 2021).

Cormorants and shags (hereafter cormorants) are a group of 41 species occurring mainly in coastal habitats throughout most of the world (del Hoyo, 2020; Nelson, 2005). They are diving visual predators, and, consequently, their foraging activities can be limited by turbidity or lack of light (Moe et al., 2021; Strod et al., 2008; van Eerden and van Rijn, 2022). While some species in highly productive regions feed on pelagic fish, most cormorants predominantly consume benthic prey (Cook et al., 2012; Nelson, 2005; Zavalaga and Paredes, 1999). As for all air-breathing diving predators, the depths they can exploit efficiently are limited by body oxygen storage, which increases with body mass (Quintana et al., 2007; Schreer and Kovacs, 1997). Hence, the benthic habitats and prey cormorants can exploit will be heavily influenced by their body size (Wilson et al., 2008).

The black-faced cormorant (*Phalacrocorax fuscescens*) is the only Australian endemic cormorant species (Nelson, 2005). Its breeding distribution ranges across the southern coast of Australia, where it forms colonies on offshore islands ranging in size from a couple of birds to more than thousand breeding pairs (Marchant et al., 1990), and forages exclusively in the marine environment (Nelson, 2005). The majority of the species breeds in south-eastern Australia, where breeding occurs in winter, whereas elsewhere within its range it breeds in spring/summer (Brothers, 2001; Marchant et al., 1990; Taylor et al., 2013). While winter is generally considered a period of increased nutritional stress for seabirds (Fort et al., 2009; Poupart, 2019), adopting a winter breeding strategy by black-faced cormorants in the region may be an adaptation for avoidance of heat stress (Cook et al., 2020), reduced local prey availability (Taylor et al., 2013) or avoidance of competition with highly abundant local species such as short-tailed shearwaters (*Ardenna tenuirostris*) (Poupart, 2019; Skira, 1991) in summer.

The black-faced cormorant is sexually dimorphic, with average male and female body masses of 1.70 kg and 1.55 kg, respectively (Riordan and Johnston, 2013). Such sexual dimorphism has been shown to be associated with sexual segregation of foraging niches in seabirds (González-Solís et al., 2000; Quillfeldt et al., 2011). In particular, as body mass can influence diving performance, the lighter mass of females may limit their foraging areas and types of prey they can access (Quillfeldt et al., 2011; Wilson et al., 2008). This could potentially impact the ability of the population to adapt to habitat alterations or restrictions in prey availability (Harris et al., 2014).

The south-eastern Australia marine region is experiencing rapid oceanic warming (Hoegh-Guldberg et al., 2019; Poloczanska et al., 2012) and the anticipated climate change impacts will lead to alterations in prey diversity, abundance and distribution (Johnson et al., 2011; Perry et al., 2005). Knowledge of the at-sea movements and habitat-use, and the factors influencing them, in the region's marine predators is vital for predicting how these species, the prey populations they depend on and the marine ecosystem they dominate may respond to environmental change (Chambers et al., 2011; Grémillet and Boulinier, 2009; Wernberg et al., 2011). However, relatively little is known of the diet, and there is currently no information on the diving behaviour and habitat-use in the black-faced cormorant, such that it is not possible to assess how such changes may affect its population trajectory (Grémillet and Boulinier, 2009; Taylor et al., 2013).

The aims of the present study, therefore, were to determine: (1) diet; (2) diving behaviour, at-sea movements and habitat-use; and (3) extrinsic and intrinsic factors influencing these parameters in black-faced cormorants.

## RESULTS

### Diet

Regurgitate samples were obtained from 79 individuals (2020: 17; 2021: 17; 2022: 23; 2023: 22; Table 1). Only one sample was obtained from each of these individuals. Identifiable remains were present in 68 (86%) of these samples. A total of 186 prey belonging to 24 species/groups were identified from the regurgitates, with all but four being fish (Table 1). Prey were almost exclusively classified as benthic, with redbait (*Emmelichthys nitidus*) and Clupeiformes sp. as the only identifiable pelagic prey species. Redbait was observed only in 2022 while Clupeiformes were only observed in 2023.

**Table 1.**
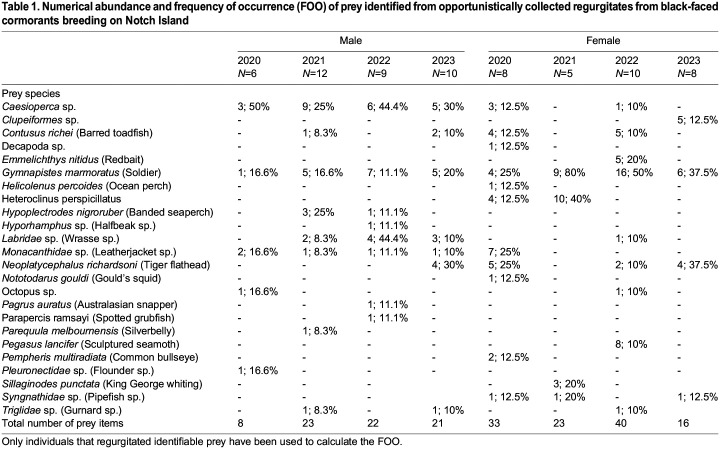
Numerical abundance and frequency of occurrence (FOO) of prey identified from opportunistically collected regurgitates from black-faced cormorants breeding on Notch Island

Most prey species had both a low numerical abundance and a low frequency of occurrence (Table 1). However, some species such as soldier (*Gymnapistes marmoratus*; 32.1±8.2% across years) and *Caesioperca* sp. (21.5±6.7% across years) were relatively frequently observed prey. For these species, soldier had a relatively high frequency of occurrence (FOO) in the diet of females (48.1±11.8% across years), whereas *Caesioperca* sp. had a relatively high FOO in male diets (37.3±5.9% across years). A linear mixed model indicated a trend for males to consume larger prey items (12.43±0.47 cm) than females (9.63±0.40 cm) but the difference between them was not significant (*P*=0.24, 95% CI: −0.36–3.73).

### Foraging trip characteristics and diving behaviour

**Fig. 1.**
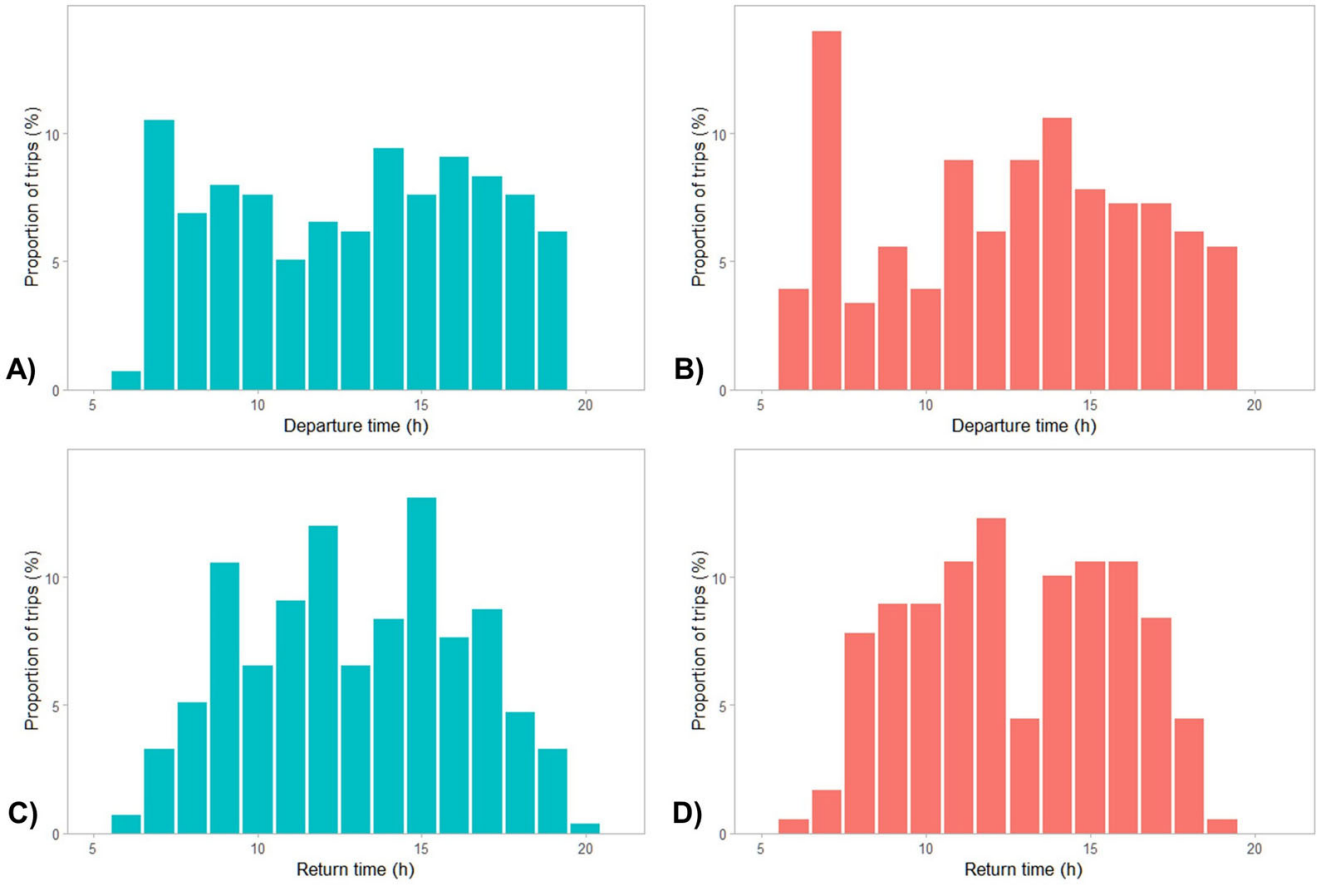
Foraging trip departure times for (A) males and (B) females and foraging trip return times for (C) males and (D) females for black-faced cormorants breeding at Notch Island.

A total of 62 loggers was deployed. However, due to device malfunction, data were obtained from 56 individuals (28 M, 28 F) across the 4 years of the study (Table 2). Data were collected for 135.5±9.5 h. A total of 64,732 dives were recorded over 455 foraging trips with a mean trip duration of 11.5±0.6 h. Mean trip durations varied substantially between years (2020: 9.1±0.7 h; 2021: 18.1±2.2 h; 2022: 11.0±1.1 h; 2023: 11.0±1.1 h) but not between sexes (M: 12.4±1.0 h, F: 10.8±0.7 h). However, the linear mixed model indicated that none of these differences were significant. Birds departed from, and returned to, the colony on foraging trips throughout the day (Fig. 1). Departures peaked around sunrise (07:00 h) while returns peaked around 12:00 h and then again at 15:00 h. While on a foraging trip, birds regularly roosted away from the colony overnight. This behaviour was observed in 184 of the 455 recorded trips and for 49 of the 56 individuals (26 M, 23 F).

**Table 2.**
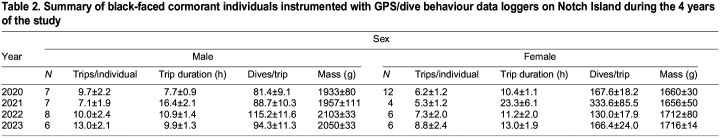
Summary of black-faced cormorant individuals instrumented with GPS/dive behaviour data loggers on Notch Island during the 4 years of the study

**Fig. 2.**
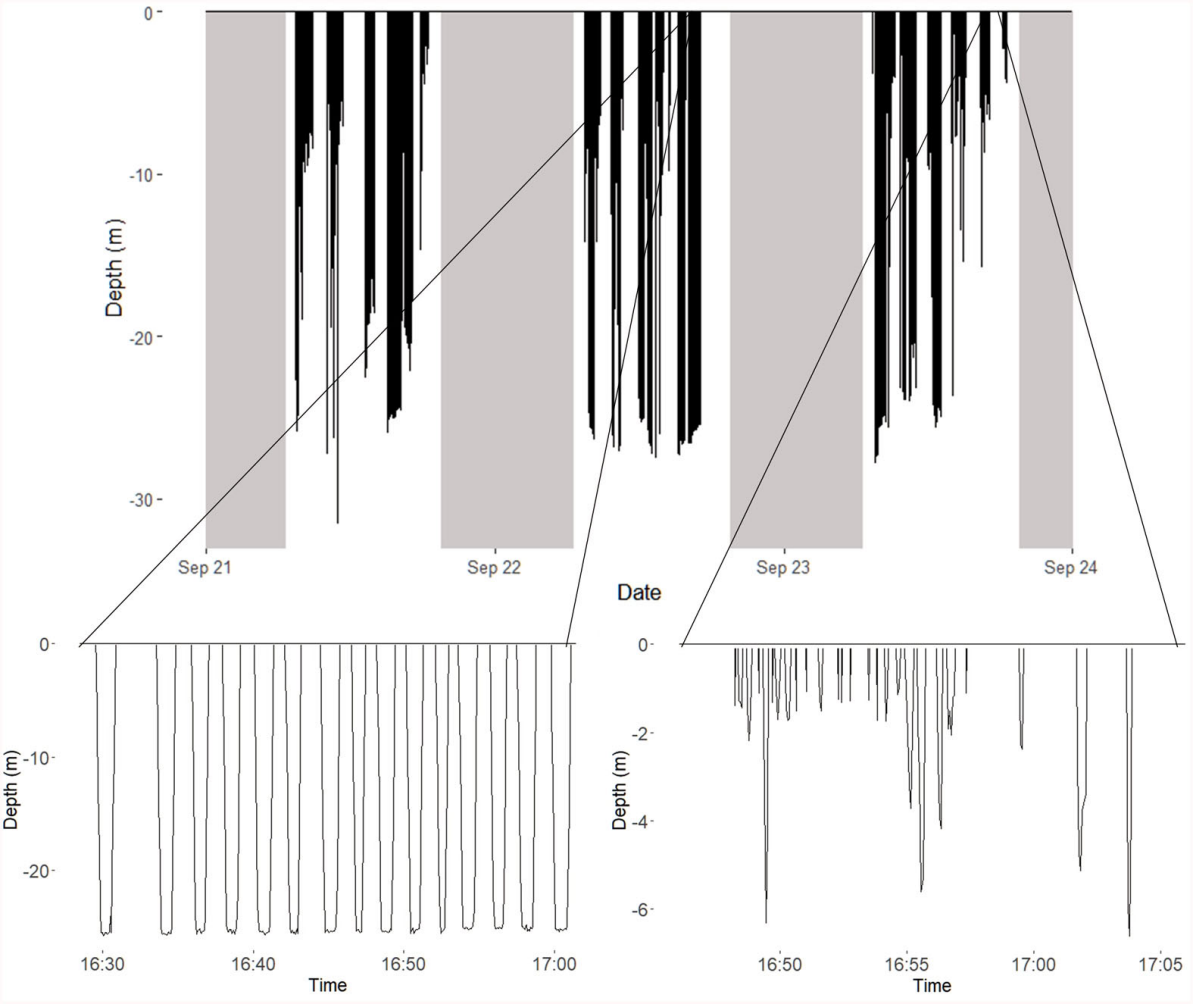
**A representative plot showing the diving data collected for a male black-faced cormorant collected over three consecutive days.** The upper panel shows the temporal distribution of diving activity. Both benthic (lower left) and pelagic (lower right) dives were observed in this individual.

**Fig. 3.**
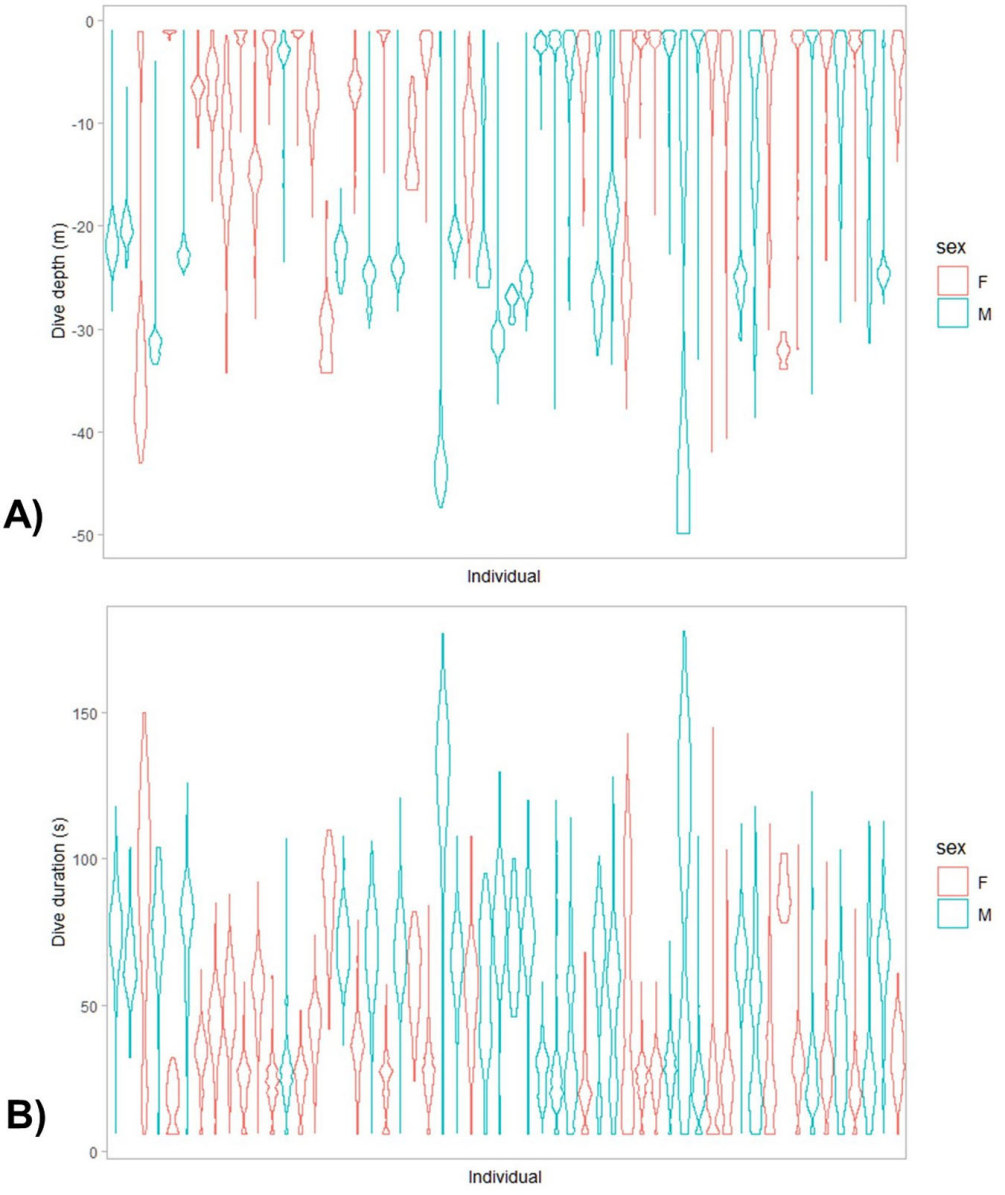
Dive depths (A) and durations (B) for black-faced cormorants breeding on Notch Island over the 4 years of study.

Diving was restricted to daylight hours (Fig. 2). While diving occurred throughout daylight hours, the generalized additive mixed model (GAMM), which investigated the vertical distance travelled throughout the day indicated that both sexes displayed greater foraging activity during the morning, with males displaying greater effort and for longer (Fig. S1). Analysis of dive profiles indicated that the majority of dives (96%) were benthic (Fig. 3). However, this proportion varied between years (2020: 1.1%; 2021: 2.0%; 2022: 7.5%; 2023: 4.9%). While most trips were exclusively benthic, 19 trips reached relatively high amounts of pelagic diving (range: 27.3%-70.5%; 2022: 14 trips; 2023: 5 trips). Dive depths and durations, collected over multiple trips, were skewed within individuals (median depth range: 1.2-43.4 m; median duration range: 17–128 s) (Fig. 3). Model averaging of the linear mixed model with dive depth as a dependent variable revealed dive depth was influenced by sex (males reaching greater maximum depths than females) and year (shallower depths in 2022; Table 3; Table S1). Model averaging of the linear mixed model with dive duration as a dependent variable revealed that dive duration increased significantly with dive depth but was not influenced by any other factors (Table 3).

**Table 3.**
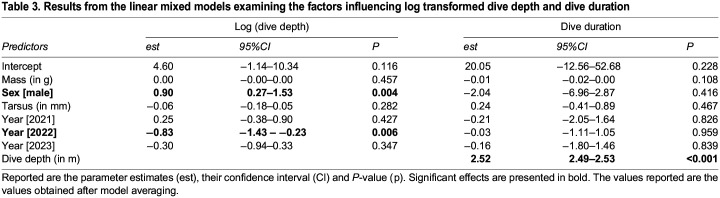
Results from the linear mixed models examining the factors influencing log transformed dive depth and dive duration

Analysis of post-dive surface duration in relation to dive duration provided evidence of an aerobic dive limit in 17 individuals (8 males, 9 females). In the remaining individuals, no rapid increase in post-dive duration was observed over the dive durations recorded. The observed aerobic dive limit was 54±6 s (range: 29-124 s). While it was not possible to ascertain whether the aerobic dive limit was different between sexes due to the small sample size, a linear model indicated it was significantly correlated with body mass (*r*^2^=0.26, *P*=0.03) (Fig. S2).

### Spatial distribution of foraging effort

**Fig. 4.**
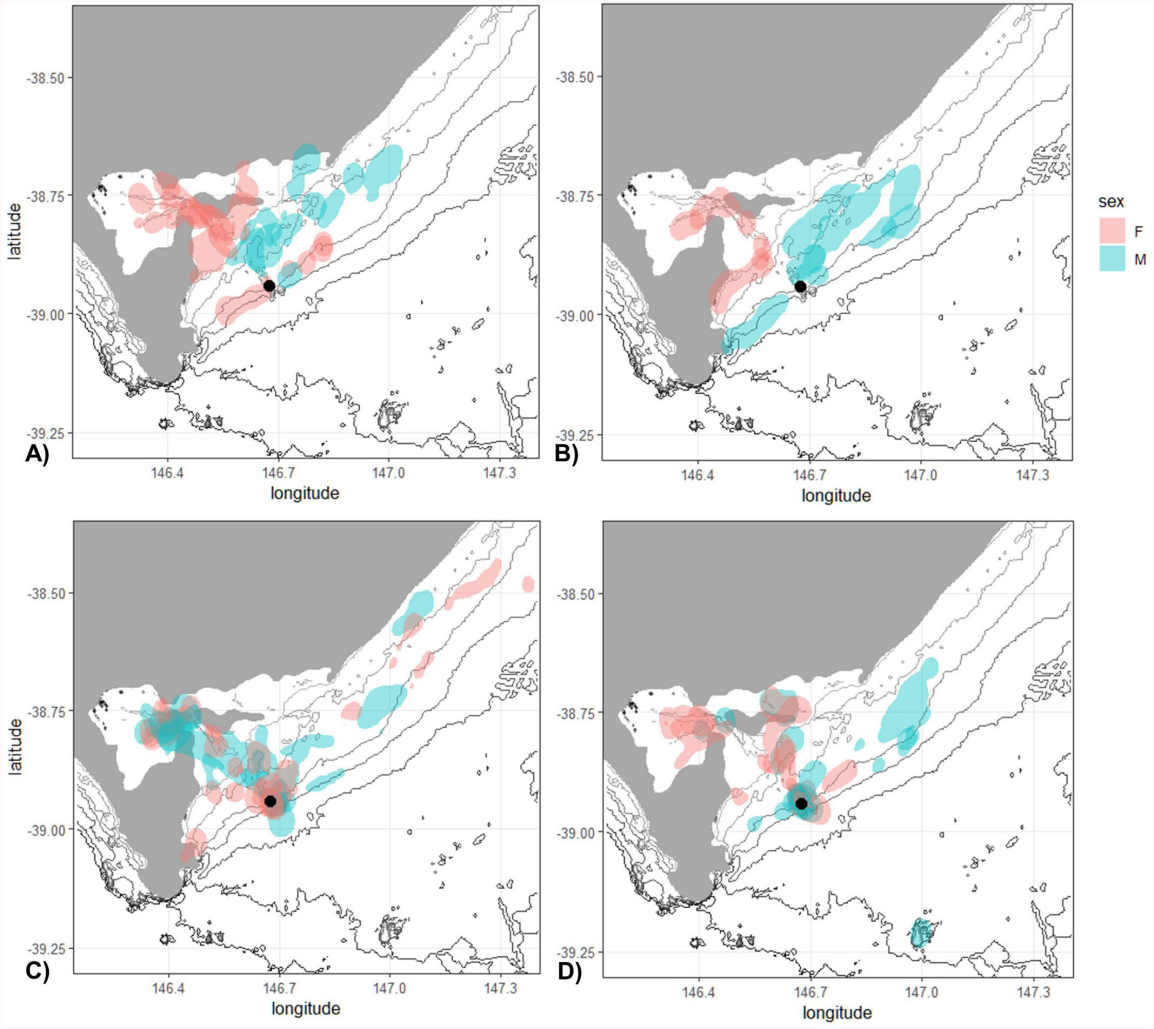
The 95% kernel UD for all diving locations for black-faced cormorants breeding on Notch Island in 2020 (A), 2021 (B), 2022 (C) and 2023 (D).

The distribution of diving locations was almost exclusively segregated by sex in 2020 and 2021 (Fig. 4). Females foraged in shallow areas (1-15 m), with a strong use of the areas in Corner Inlet, a shallow bay to the northwest of the colony, while males mainly foraged in deeper areas (20-40 m). The overlap in foraging locations between males and females differed between years (2020: 0.10%; 2021: 0.02, 2022: 0.85; 2023: 0.72). The high degree of overlap in 2022 was associated with an increase in males foraging in or near Corner Inlet and an increase in females foraging closer to the breeding colony, where they made primarily pelagic dives (Fig. 4). While sexual segregation in 2023 was stronger than in 2022, some males still foraged in shallower areas and some females foraged in deeper areas around the colony where pelagic dives were observed (Fig. 4).

## DISCUSSION

Despite its wide distribution, relatively little is known of the foraging ecology of the black-faced cormorant, an Australian endemic. Such information is urgently required in view of the rapid oceanic warming of the species' south-eastern Australia distribution. The present study investigated the diet, diving behaviour and habitat-use of black-faced cormorants during chick-rearing. Observed prey species were almost exclusively benthic, consistent with the recorded dive behaviour. Males foraged at deeper depths than females, presumably due to a greater physiological diving capacity derived from their larger size. This was associated with sexual segregation of foraging locations, with females predominantly frequenting the shallower areas closer to the coastline.

### Diet and foraging behaviour

While a high prey diversity was observed in the present study, most species were recorded infrequently, with only a couple of species being found more commonly. These findings, consistent with those of Taylor et al. (2013), indicate that the black-faced cormorant is a generalist forager on the population level. While no significant sex differences were observed, there was a tendency for some prey species to be observed more frequently in one sex, potentially reflecting differences in foraging areas and/or depth. Despite inter-annual differences in the proportion of pelagic dives, no significant differences in prey size or species composition could be detected between years. This lack of differences was likely caused by the opportunistic nature of our sampling, which used voluntarily regurgitated samples that can be biased towards the most recently consumed or larger, slower-digested items (Barrett et al., 2007). Nonetheless, the prey species observed are largely consistent with those previously recorded by Taylor et al. (2013) in regurgitate pellets at the same study site.

Black-faced cormorants were observed to leave for foraging trips throughout daylight hours. While there was a peak in departures during the morning, departures occurred constantly throughout the remainder of the day. Returns from foraging trips were relatively constant throughout the day, but showed a slight dip around midday, which was accompanied by two peaks before and afterwards. The observed peak in departures around sunrise is consistent with the observation that cormorants in this study were only observed to dive during daylight hours. The restriction of diving activity to primarily daylight hours is consistent with cormorants being visual predators (Martin et al., 2008; Moe et al., 2021; Strod et al., 2008). Leaving around sunrise likely allows individuals to invest as much time as possible in foraging, which is especially important during the highly demanding chick rearing period. Individuals captured for device deployment were in the post-brooding stage, decreasing the constraints posed by nest attendance. For some species of marine cormorants, sex-specific differences in the timing of diving have been observed (Harris et al., 2013; Wanless et al., 1995), but this was not the case for black-faced cormorants.

While on foraging trips, black-faced cormorants were regularly observed to roost on islands and islets throughout their foraging range, and often overnight. This behaviour was observed for both sexes and for the majority of the individuals. Overnight roosting away from the colony might confer energetic savings by not having to fly back to the colony when not enough food has been acquired to cover the energetic demands of the parent or the chick. However, further investigation is required to determine the factors influencing this behaviour and the effect on foraging efficiency.

While there was a high variation in dive depths at a population level, dive depths, which were recorded over multiple foraging trips, were strongly skewed within individuals. Skewed dive depths and repeatedly exploiting the same depths has been observed in a range of cormorant species where it has been linked to individual foraging specialisation (Camprasse et al., 2017a; Cook et al., 2006; Morgan et al., 2019). Dive depths and durations were found to be comparable to other similar-sized cormorants (Quintana et al., 2007).

Dive depth was found to be significantly deeper in males than females. Sexual segregation in dive depths between sexes has been found in a range of cormorant/shag species and has been linked to sexual dimorphism (Cook et al., 2013; Kato et al., 1999; Quintana et al., 2011). In comparison to other years, for both sexes, the dive depth was significantly lower in 2022, a year when a relatively high proportion of pelagic diving was observed in comparison to other years. Dive duration increased with dive depth, a feature common in all diving species (Boyd, 1997; Quintana et al., 2007), and consequently dive duration was significantly greater in males than females.

The observed sex differences in maximum dive depth and duration in the present study are likely due to differences in their aerobic dive limit. While no significant difference in aerobic dive limit was observed between the sexes, potentially due to low sample size, there was a positive relationship between aerobic dive limit and body mass. This is consistent with the hypothesis that larger animals have greater body oxygen stores, enabling greater dive durations and, thus, deeper dive depths (Schreer and Kovacs, 1997). As black-faced cormorants display sexual dimorphism (Riordan and Johnston, 2013), the heavier males would be expected to have greater physiological diving capacity, enabling foraging at greater depths and for longer than females. This potentially enables heavier individuals to forage in areas with less competition or with better food availability.

While black-faced cormorants in the present study were predominantly benthic divers, pelagic dives were also observed, and their prevalence differed between years. While pelagic dives were rare in 2020, 2021 and 2023 (<2%), they were more frequent in 2022 and 2023 (respectively 7.5% and 4.9%). During the 2022 data collection period, juvenile humpback whales (*Megaptera novaeangliae*) were regularly observed foraging near the colony in mixed species aggregations comprising of dolphins (*Tursiops sp.* and *Delphinus sp.*), Australasian gannets (*Morus serrator*), terns (*Thalasseus sp.*), pacific gulls (*Larus pacificus*), silver gulls (*Chroicocephalus novaehollandiae*) and black-faced cormorants (pers. obs.). These observations suggest a concentrated, high abundance of pelagic schooling prey in the region which may have enabled black-faced cormorants to predate on these opportunistically.

### Segregation in spatial distribution of diving

The spatial distribution of diving locations for black-faced cormorants showed a near total separation between male and female foraging habitats in 2020, 2021 and 2023. This segregation in foraging areas could be linked to differences in nutritional needs (Lewis et al., 2002). However, in the present study, this separation was strongly linked to seafloor depth, likely a consequence of a greater aerobic dive limit in males enabling them to forage at deeper depths. Although some of the females foraged at deeper depths, these were larger individuals. The deeper regions where the males foraged are characterised by a seafloor which mainly consists of sand and gravel (Lucieer et al., 2019). In contrast, females mainly foraged in Corner Inlet, a shallow bay to the northwest of the colony which consists of extensive shallow mudflats covered with seagrass, as well as some deeper channels which run through Corner Inlet between the seagrass beds (Lucieer et al., 2019). The different regions and associated habitats used by both sexes likely explain the higher prevalence of some prey species in either male or female diets.

Surprisingly, in 2022, a year which was also characterised by a higher proportion of pelagic diving, there was a high overlap in foraging habitat used by males and females. This overlap, in combination with the higher proportion of pelagic diving, suggests that a change has occurred in prey availability. However, as no data on fish distribution or abundance are available for the region, it is not possible to determine which changes may have occurred.

Currently, the factors influencing prey availability for black-faced cormorants are unknown. However, for Australian fur seals in south-eastern Australia, which are also predominantly benthic foragers, lagged effects of the Indian ocean dipole (IOD) have been found on foraging, where positive values resulted in more efficient benthic foraging, and negative values resulted in a higher proportion of pelagic prey species (Speakman et al., 2021). The IOD for 2022 was negative (Bureau of Meteorology, 2022), which could influence prey availability and the resulting foraging behaviour of black-faced cormorants. However, further studies are required to unravel the relationships between large-scale climatic indices and foraging behaviour and distribution in black-faced cormorants.

While for some cormorant species males and females forage in the same areas (Camprasse et al., 2017b), the spatial segregation in foraging habitat observed in the present study is consistent to that found for other cormorant species (Fijn et al., 2022; Quintana et al., 2011; Soanes et al., 2014). However, in the present study, the degree of sexual segregation varied between years. Similar inter-annual variation in foraging distribution has been observed in Imperial cormorants (*Leucocarbo atriceps*), likely due to environmentally mediated fluctuations in prey availability or distribution (Quillfeldt et al., 2011; Quintana et al., 2022).

The segregation in foraging habitat between males and females, which was observed in 3 out of 4 years, might have implications for the reproductive success and future population trajectory of the species. Indeed, environmental changes, for example due to climate change, or anthropogenic influences, could affect resource distribution throughout the foraging range of black-faced cormorants. As a result of the observed sexual segregation in exploited habitats and areas, there might be disproportionate effects of environmental changes on resource availability to a single sex. However, as for most seabirds, both partners contribute to raising the chicks, and therefore, negative impacts on one sex could be expected to reduce reproductive success (Schreiber and Burger, 2001). While cormorants, as long-lived seabirds, are resistant to occasional breeding failure, repeated failures can be expected to eventually impact demography and population sizes (Oppel et al., 2022; Schreiber and Burger, 2001).

In summary, black-faced cormorants in this study were found to be predominantly benthic foragers. Their diving behaviour and exploited foraging depths were found to be linked to body mass, and thereby, also to their sex. These differences in diving behaviour were found to lead to the exploitation of different areas by males and females. However, the degree of sexual segregation differed between years, suggesting that the spatial foraging distribution of black-faced cormorants is not only influenced by physiology, but also by changing spatial distributions in resource availability and abundance. To adequately determine how resource distribution differs between years, which environmental factors influence this, and how this influences the foraging behaviour of black-faced cormorants, additional years of data collection are required. This might allow to predict the responses of black-faced cormorants to the expected effects of climate change.

## MATERIALS AND METHODS

### Animal handling and data collection

**Fig. 5.**
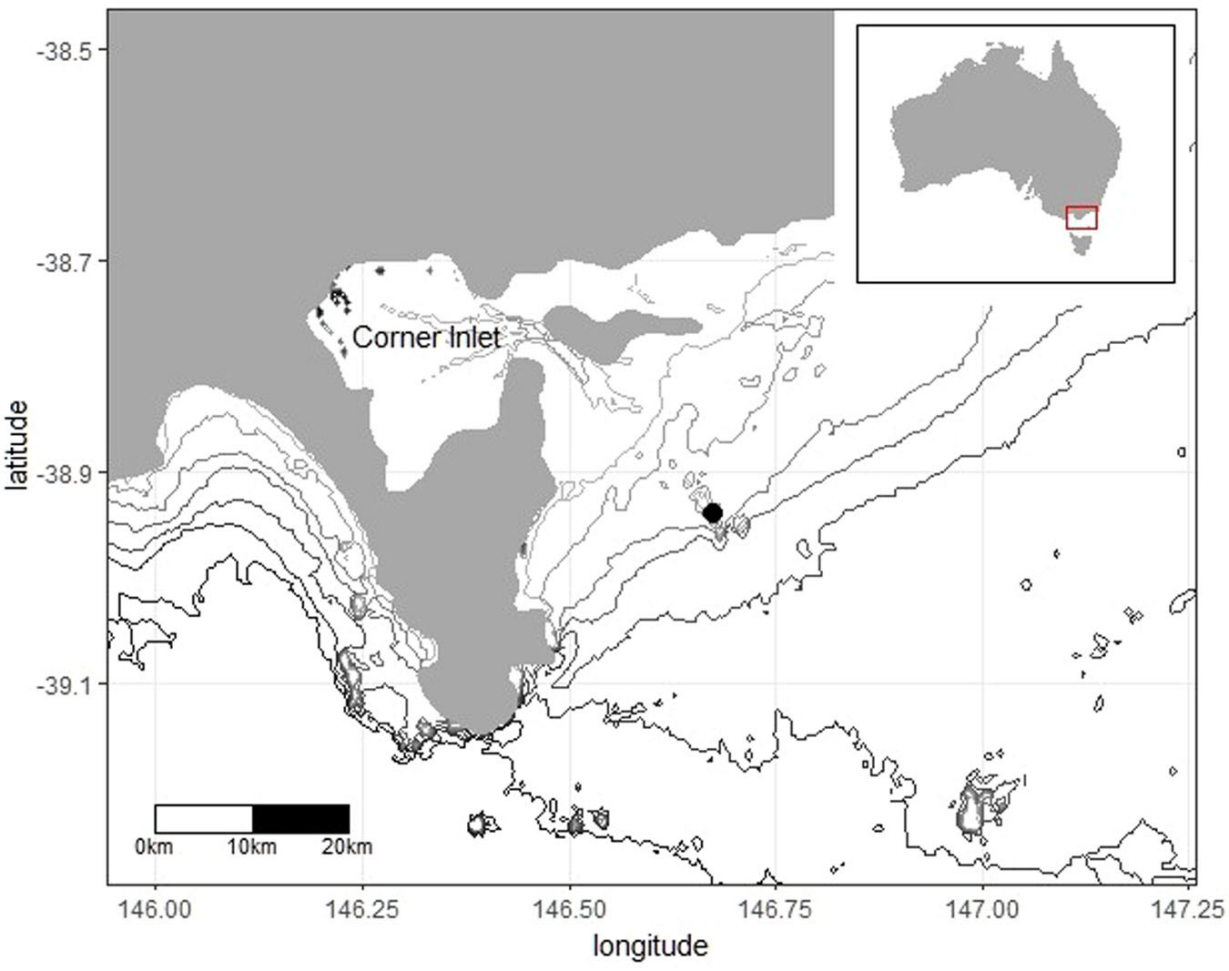
**Location of the black-faced cormorant (*Phalacrocorax fuscescens*) study colony at Notch Island (black circle) and the shallow Corner Inlet region.** Bathymetry isobaths are plotted in 10 m intervals.

The study was conducted on Notch Island (38°56′25″S 146°40′33″E) (Fig. 5), northern Bass Strait (south-eastern Australia), which hosts a black-faced cormorant colony of approximately 950 nests (Taylor et al., 2013). All animal handling procedures were in accordance with Deakin University Animal Ethics committee approvals (B12-2020, B34-2022) and Department of Energy, Environment and Climate Action (Victoria, Australia) wildlife research permits (10009521, 10010406). Access to the island was provided through a parks Victoria access agreement (AA0001127). Data collection occurred during chick-rearing (September-October) in 2020-2023. Adults rearing chicks aged approximately 20-40 d (post brooding) were captured with a noose-pole while attending the breeding colony. Individuals were weighed in a cloth bag using a spring scale (±25 g, Salter, Bristol, UK) and morphometric measurements (culmen length, bill length, tarsus length and bill depth) were taken using a vernier calliper (±0.1 mm). Two body contour feathers were collected (for molecular sexing) and a uniquely numbered metal leg band was applied to the left tarsus. A combined pressure and GPS data logger (Pathtrack nanoFix^®^ GEO+RF, 20 g, 1.1±0.02% of the body mass) was then attached to two central tail feathers with waterproof tape [TESA^®^ 4651, Beiersdorf AG, Germany (Wilson et al., 1997)] before individuals were released near the edge of the colony to resume normal behaviour (Table 2). The instrumentation procedure from catching to release lasted approximately 10-15 min.

The data logger was programmed to record pressure every 4 s, and a GPS location every 5 min (2020, 2022 and 2023) or every 10 min (2021). Data were downloaded over a UHF link to a base station near the colony until the logger battery expired or the device was shed.

If cormorants regurgitated voluntarily upon handling during device deployment, prey items were photographed and stored frozen (–20°C) for later analysis. Additional samples were collected from individuals handled for a concurrent study. In the laboratory, all prey were thawed and identified to the lowest taxonomic level possible using a relevant fish guide (Gomon et al., 2008). Whole prey items were measured with a metal ruler (±1 mm): fork length for fish; mantle length for cephalopods.

### Data processing and analyses

All data were processed and analysed in the R statistical environment (R Core Team, 2018) (version 4.1.3). The frequency of occurrence (FOO; i.e. proportion of samples in which a prey species is found) and average numerical abundance (i.e. number of times a prey is observed in each sample) were calculated separately for male and female cormorants and for each year of study. Only identifiable prey items were included when calculating FOO and numerical abundance. Due to the limited sample size, no formal tests of differences in diet composition between sexes or years was possible. Only whole prey items, for all species combined, were used to investigate the factors influencing prey length. A linear mixed model with the individual as a random factor, sex and year as fixed factors and prey length as the dependent variable was used.

The GPS tracks were split into trips using the *track2kba* package (Beal et al., 2021) with a buffer of 500 m around the colony being used as a threshold for the start and end of trips. The size of this buffer was based on the size of the island, and the observation that cormorants regularly roosted on the island away from the breeding colony. Summary statistics of trip duration (h), total trip distance (km) and maximum distance from the colony (km). To investigate the factors influencing trip duration, a linear mixed model containing sex and year of study as fixed factors, and the individual as a random factor was used. Trip duration was log-transformed to adhere to model assumptions.

The pressure data were processed using the *diveMove* package (Luque, 2007). As pressure data were only collected every 4 s, depth was linearly interpolated between the surface and the first two and last two points of each dive to calculate actual dive duration. Summary statistics of dive duration, maximum dive depth and vertical distance covered in a dive were then derived. It should be noted that, as a result of the 4 s interval, both dive depths and vertical distance were likely underestimated. Aerobic dive limit was investigated in each individual from the dive duration and post-dive duration data using the constraint lines method (Horning, 2012) (Fig. S3). For the subset of individuals where an aerobic dive limit was detected, the influence of body mass was assessed using a linear model.

Dive locations were determined by linear interpolation of the GPS tracking data using the *adehabitatLT* package (Calenge, 2011a). For each dive location the sea-floor depth was extracted from the GEBCO dataset using the *Marmap* package (Pante and Simon-Bouhet, 2013). If the maximum depth of a dive was within 10% of either the previous or subsequent dive, the bottom phase of the dive was assumed to have occurred on the seafloor and the animal adopted a benthic foraging strategy (Tremblay and Cherel, 2000). If dives did not meet this criterion, they were considered pelagic. Dive data were only collected every 4 s, which might lead to an underestimation of dive depth. Therefore, an additional criterion was used. If the bathymetric depth at the dive location was <10 m or the difference between dive depth and bathymetric depth was less than 5 m, the dive was assumed to be benthic.

To investigate the factors influencing diving behaviour, the relationships between the dependent variables of dive depth and dive duration, and predictive intrinsic and extrinsic factors were assessed. For both dependent variables, a linear mixed model was used with sex, tarsus length (as a measure of structural size), mass and year of study as fixed factors, and the individual and the trip number nested within the individual as random factors. In the model where dive depth was the dependent variable, dive depth was log-transformed to adhere to model assumptions. In the model analysing factors influencing dive duration, dive depth was included as a fixed variable to account for the fact that dive duration is expected to increase with dive depth (Boyd, 1997). The linear mixed models were generated using the *lme4* package (Bates et al., 2015). For model selection, the full model was specified, and the ‘dredge’ function in the *MuMIn* package (Barton, 2018) was used to compare all possible models. Model averaging was conducted where multiple candidate models had ΔAICc<4 to determine the factors with consistent influences on the dependent variables. Model assumptions were assessed using the ‘check_model’ function in the *performance* package (Lüdecke et al., 2021). Unless otherwise indicated, all results are reported as mean±s.e.

Temporal patterns in dive rate [m·h^−1^, vertical distance travelled, an index of foraging effort (Boyd et al., 1991)], were investigated using a GAMM with time of day (in AEDT) as the explanatory variable and the individual was used as a random factor. A circular smoother was applied for time of day, and the number of knots (*k*) was set to 24. Sex was used as a grouping factor in the model as for some cormorant species, males and females have been observed to forage at different times of day (Harris et al., 2013; Wanless et al., 1995). The GAMM was constructed using the *MGCV* package (Wood and Wood, 2015).

To analyse the spatial distribution of diving, the 95% kernel home range for all dive locations was determined using the *adehabitatHR* package (Calenge, 2011b). Kernel home ranges were calculated for each individual and subsequently plotted for each year and sex separately to determine whether these influenced the spatial distribution. The smoothing factor (*h*) was set to 0.01. For each year of study, the overlap between male and female kernel home ranges of diving locations was determined using Bhattacharyya's Affinity Index which ranges from 0, indicating no overlap, to 1, indicating complete overlap. This was calculated using the *adehabitatHR* package (Calenge, 2011b).

## Supplementary Material

10.1242/biolopen.060336_sup1 Supplementary information
