# Supplementary material for: Foraging behaviour and habitat use during chick-rearing in the Australian endemic black-faced cormorant (Phalacrocorax fuscescens)
Source: Biol Open. 2024 May 16;13(5):bio060336. doi: 10.1242/bio.060336 (PMC11128270; doi:10.1242/bio.060336)
Supplement: Supplementary information [file biolopen-13-060336-s1.pdf]

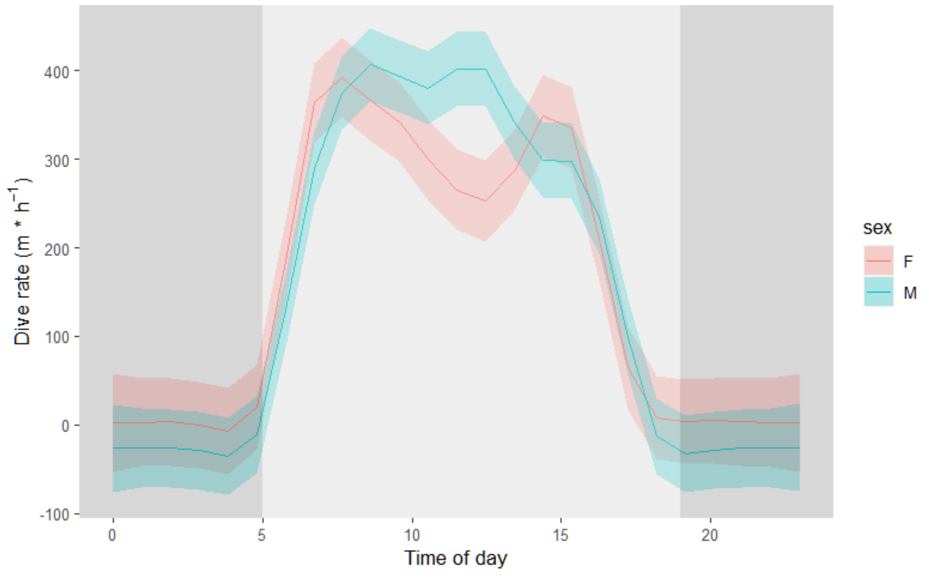

Fig. S1. GAMM predicted variation in dive rate (m·h^{-1}) during the day for all individuals. The shaded areas indicate the 95% CI. Approximate time of daylight is indicated in light grey. Time of day is reported in AEDT (Australian eastern daylight time).

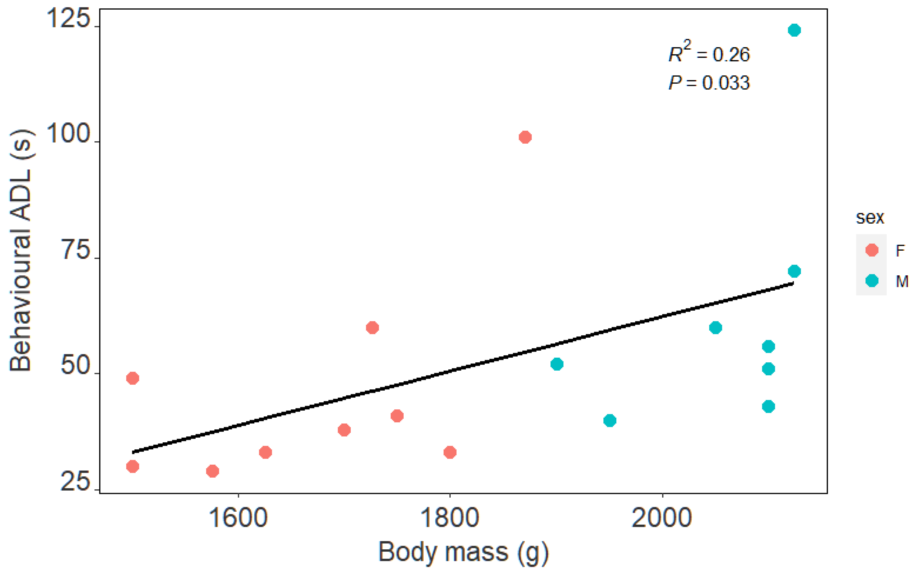

Fig. S2. The relationship between behavioural aerobic dive limit (s) and body mass (g) for black-faced cormorant individuals where an aerobic dive limit was detected. The black line indicates model predicted values derived from a linear model containing behavioural ADL as a dependent variable and body mass as a predictor variable.

Biology Open • Supplementary information

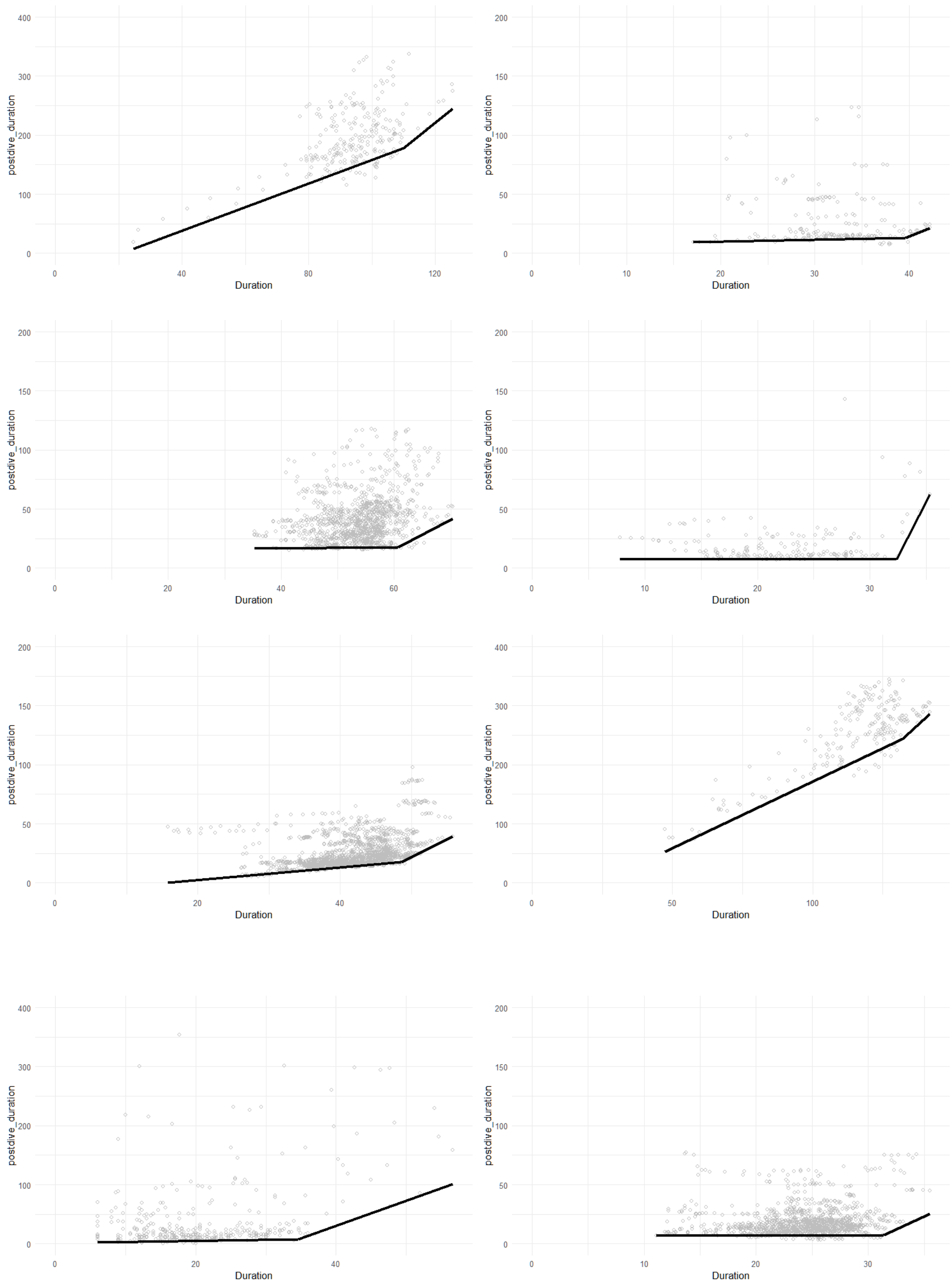

Biology Open • Supplementary information

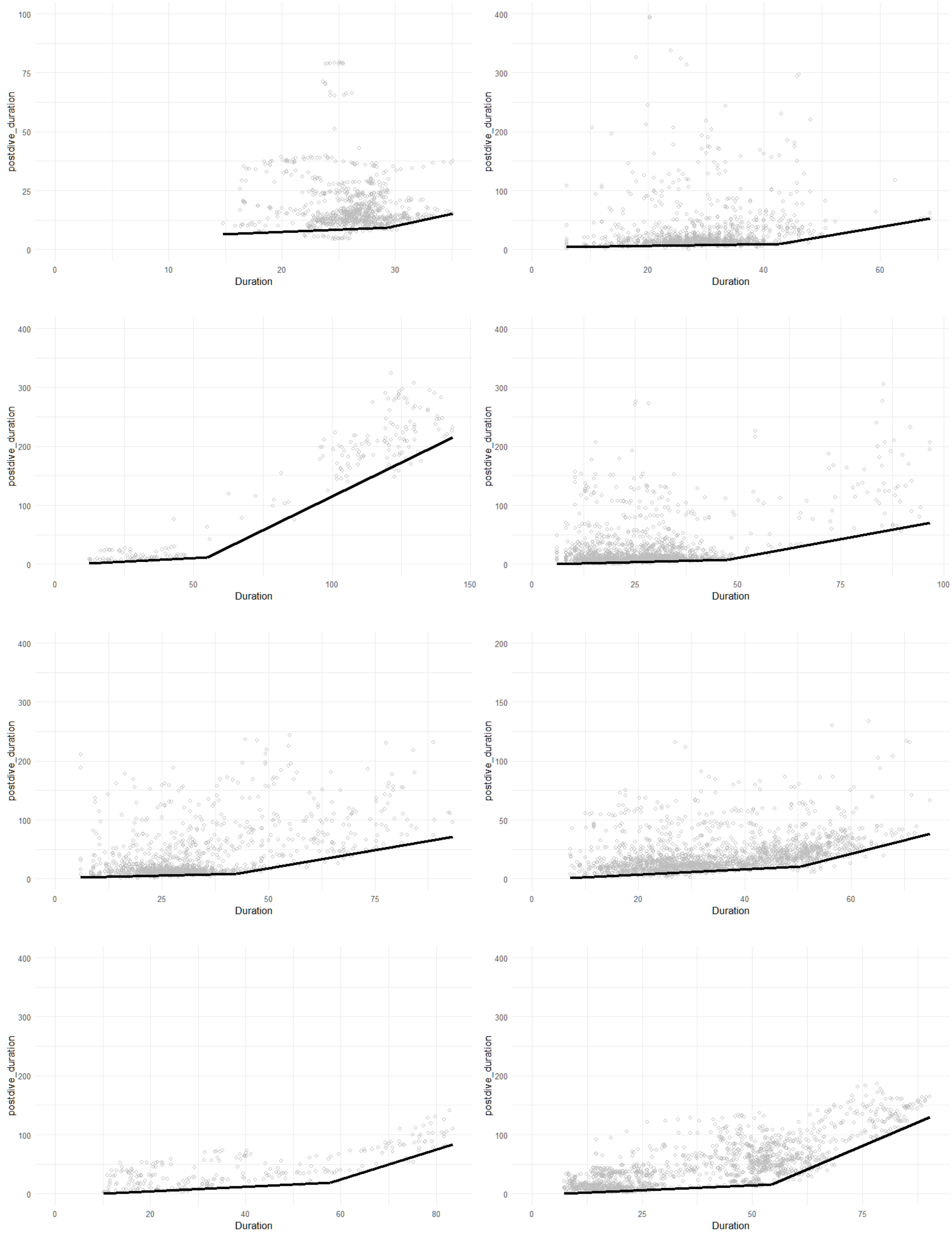

Biology Open • Supplementary information

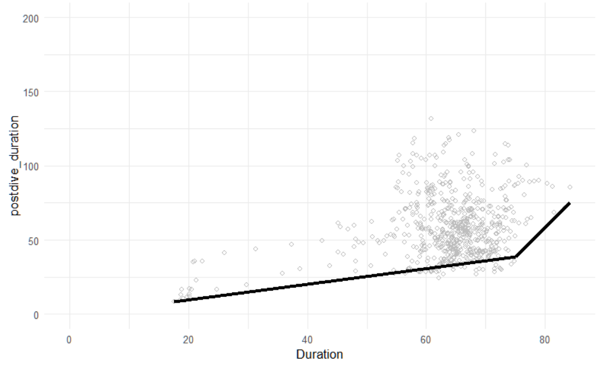

Fig. S3. Plots with constraint lines used to determine the aerobic dive limit for all individuals where this was detected. The intersection of the black lines indicates the behavioural aerobic dive limit.

Biology Open • Supplementary information

Table S1. The candidate models for which model averaging was applied for log transformed dive depth and dive duration. Linear mixed models were used, and the full models were Log (dive depth) ~ Intercept + mass + sex + tarsus + year and dive duration ~ Intercept + mass + sex + tarsus + year + dive depth. The models for log(dive depth) and dive duration included the individual and trip ID nested in within the individual as random factors.

rank	Candidate models	df	LogLik	AICc	ΔAICc	Weight
	Log (dive depth) ~					
1	Intercept + mass + sex + tarsus + year	10	-48831.9	97683.7	0.00	0.370
2	Intercept + sex + tarsus + year	9	-48833.3	97684.7	0.96	0.229
3	Intercept + sex + year	8	-48834.6	97685.2	1.44	0.180
4	Intercept + mass + sex + year	8	-48834.3	97686.6	2.89	0.087
	Dive duration~					
1	Intercept + mass + sex + tarsus + dive depth	8	-224604.8	449225.5	0.00	0.200
2	Intercept + mass + dive depth	6	-224606.8	449225.6	0.09	0.191
3	Intercept + mass + tarsus + dive depth	7	-224605.8	449225.6	0.11	0.189
4	Intercept + mass + sex + dive depth	7	-224606.0	449226.0	0.46	0.159
5	Intercept + sex + dive depth	6	-224607.5	449226.9	1.37	0.101
6	Intercept + sex + tarsus + dive depth	7	-224607.4	449228.7	3.17	0.041
7	Intercept + mass + tarsus + year + dive depth	10	-224605.5	449229.0	3.42	0.036
8	Intercept + mass + sex + tarsus + year + dive depth	11	-224603.6	449229.2	3.64	0.032
